# Mechanisms of CNS Viral Seeding by HIV^+^ CD14^+^ CD16^+^ Monocytes: Establishment and Reseeding of Viral Reservoirs Contributing to HIV-Associated Neurocognitive Disorders

**DOI:** 10.1128/mBio.01280-17

**Published:** 2017-10-24

**Authors:** Mike Veenstra, Rosiris León-Rivera, Ming Li, Lucio Gama, Janice E. Clements, Joan W. Berman

**Affiliations:** a Department of Pathology, Albert Einstein College of Medicine, Bronx, New York, USA; b Department of Molecular and Comparative Pathobiology, Johns Hopkins University School of Medicine, Baltimore, Maryland, USA; c Vaccine Research Center, National Institute of Allergy and Infectious Diseases, Bethesda, Maryland, USA; d Department of Microbiology and Immunology, Albert Einstein College of Medicine, Bronx, New York, USA; Harvard School of Public Health

**Keywords:** CNS, HIV, cognition, qPCR, reservoirs

## Abstract

HIV reservoirs persist despite antiretroviral therapy (ART) and are established within a few days after infection. Infected myeloid cells in the central nervous system (CNS) may contribute to the establishment of a CNS viral reservoir. The mature CD14^+^ CD16^+^ monocyte subset enters the CNS in response to chemokines, including CCL2. Entry of infected CD14^+^ CD16^+^ monocytes may lead to infection of other CNS cells, including macrophages or microglia and astrocytes, and to release of neurotoxic early viral proteins and additional cytokines. This contributes to neuroinflammation and neuronal damage leading to HIV-associated neurocognitive disorders (HAND) in ~50% of HIV-infected individuals despite ART. We examined the mechanisms of monocyte entry in the context of HIV infection and report for the first time that HIV^+^ CD14^+^ CD16^+^ monocytes preferentially transmigrate across the blood-brain barrier (BBB). The junctional proteins JAM-A and ALCAM and the chemokine receptor CCR2 are essential to their preferential transmigration across the BBB to CCL2. We show here that JAM-A and ALCAM are increased on HIV^+^ CD14^+^ CD16^+^ monocytes compared to their expression on HIV^exp^ CD14^+^ CD16^+^ monocytes—cells that are uninfected but exposed to HIV, viral proteins, and inflammatory mediators. Antibodies against JAM-A and ALCAM and the novel CCR2/CCR5 dual inhibitor cenicriviroc prevented or significantly reduced preferential transmigration of HIV^+^ CD14^+^ CD16^+^ monocytes. This indicates that JAM-A, ALCAM, and CCR2 may be potential therapeutic targets to block entry of these infected cells into the brain and prevent or reduce the establishment and replenishment of viral reservoirs within the CNS.

## IMPORTANCE

HIV infects different tissue compartments of the body, including the central nervous system (CNS). This leads to establishment of viral reservoirs within the CNS that mediate neuroinflammation and neuronal damage, contributing to cognitive impairment. Our goal was to examine the mechanisms of transmigration of cells that contribute to HIV infection of the CNS and to continued replenishment of CNS viral reservoirs, to establish potential therapeutic targets. We found that an HIV-infected subset of monocytes, mature HIV^+^ CD14^+^ CD16^+^ monocytes, preferentially transmigrates across the blood-brain barrier. This was mediated, in part, by increased junctional proteins JAM-A and ALCAM and chemokine receptor CCR2. We show that the CCR2/CCR5 dual inhibitor cenicriviroc and blocking antibodies against the junctional proteins significantly reduce, and often completely block, the transmigration of HIV^+^ CD14^+^ CD16^+^ monocytes. This suggests new opportunities to eliminate infection and seeding or reseeding of viral reservoirs within the CNS, thus reducing neuroinflammation, neuronal damage, and cognitive impairment.

## INTRODUCTION

Antiretroviral therapy (ART) has improved the life span of HIV-infected individuals by successful suppression of viral replication (1–3). However, ART does not eliminate infected cells harboring nonreplicating virus or viral reservoirs, and even after years of viral suppression, discontinuation of ART can lead to reactivation of viral replication (4, 5). Seeding of viral reservoirs occurs within 3 to 5 days after peripheral infection (6, 7). Once established, viral reservoirs persist in many different body compartments, including the gut and central nervous system (CNS) (8). Although latently infected resting CD4^+^ T cells are recognized as the major cell reservoir, HIV enters the CNS within 4 to 8 days after peripheral infection, likely by entry of infected monocytes that contribute to the establishment and maintenance of the CNS viral reservoir (9–14). Within the CNS, monocytes/macrophages and microglia are the major cell types that contain replication-competent virus, even after long-term viral suppression by ART (15–17).

Entry of HIV-infected and uninfected monocytes into the CNS is mediated, in part, by chemokines such as CCL2, which is constitutively expressed and also increased in the brain of infected individuals despite ART (18–22). After HIV-infected monocytes enter the brain, they may differentiate into macrophages and/or release infectious virus that can infect other CNS cells, including perivascular macrophages and microglia and, to a lesser extent, astrocytes (23, 24). Infected CNS cells will elaborate additional inflammatory mediators and, despite ART, produce early viral proteins such as Tat and gp120, which contribute to neuroinflammation and neuronal damage (25–30). These ongoing insults may lead to HIV-associated neurocognitive disorders (HAND) (31–33), which manifests despite ART in ~50% of infected individuals, even in those with undetectable viral loads (27, 34–36). HAND encompasses a spectrum of disease severities, including asymptomatic neurocognitive impairment (ANI), mild neurocognitive disorder (MND), and HIV-associated dementia (HAD). The prevalence of ANI and MND has increased in the ART era to approximately 30 and 20%, respectively, while that of HAD has decreased from 20% to 5% of infected people. However, even individuals diagnosed with ANI and MND experience impairment in at least 2 neurocognitive domains, including difficulties in activities of daily living for those with MND (27).

Both pre- and post-ART, CD14^+^ CD16^+^ monocytes have been extensively associated with HAND and all of these disorders’ encompassing severities (11, 12, 22, 37–42). This mature monocyte subset that expresses both the lipopolysaccharide (LPS) receptor CD14 and the FcγIII receptor CD16 is increased in number in the peripheral blood of HIV-infected individuals. It is the monocyte population most permissive to HIV infection, as well as the one that preferentially transmigrates across the blood-brain barrier (BBB) (21, 37, 43–45). We showed previously that the junctional proteins JAM-A and ALCAM are essential to transmigration of CD14^+^ CD16^+^ monocytes (21, 46). We also demonstrated in another study that CCR2, the only known receptor for CCL2 on monocytes, is increased on the surface of infected CD14^+^ CD16^+^ monocytes and that it mediates their increased sensitivity and transmigration to CCL2 (21, 38). Infected CD14^+^ CD16^+^ monocytes, like all cell types in HIV-infected individuals, are heterogeneously infected. They consist of cells that are infected with HIV (HIV^+^ CD14^+^ CD16^+^ monocytes) and cells that remain uninfected but that are exposed to virus, early viral proteins, and inflammatory mediators (HIV^exp^ CD14^+^ CD16^+^ monocytes). It remains unclear to what extent HIV^+^ CD14^+^ CD16^+^ monocytes cross the BBB, and we propose that blocking the entry of HIV^+^ CD14^+^ CD16^+^ monocytes into the CNS will eliminate, or limit, establishment and replenishment of viral reservoirs, which subsequently may reduce neuroinflammation and HAND.

To characterize the mechanisms of viral infection of the CNS and to determine potential therapeutic targets that may limit the infection and continued replenishment of CNS viral reservoirs by HIV^+^ CD14^+^ CD16^+^ monocytes, we examined their transmigration across an *in vitro* model of the human BBB. We show that HIV^+^ CD14^+^ CD16^+^ monocytes preferentially transmigrate across the BBB and that this is mediated in part by increased surface JAM-A and ALCAM. We also show that antibodies against JAM-A and ALCAM eliminate or significantly reduce transmigration of HIV^+^ CD14^+^ CD16^+^ monocytes. In addition, we demonstrate that CCR2, when targeted with the novel CCR2/CCR5 dual inhibitor cenicriviroc (CVC) (47), may also be a therapeutic target to eliminate or reduce transmigration of HIV^+^ CD14^+^ CD16^+^ monocytes. These data suggest that initial seeding and continued reseeding of the CNS viral reservoir may be prevented or significantly reduced by adding these antibodies, the inhibitor, or a combination thereof to an ART regimen or to preexposure prophylaxis and that this as well will reduce neuroinflammation, neuronal damage, and HAND.

## RESULTS

### HIV infection and HIV exposure of mature CD14^+^ CD16^+^ monocytes.

**FIG 1:**
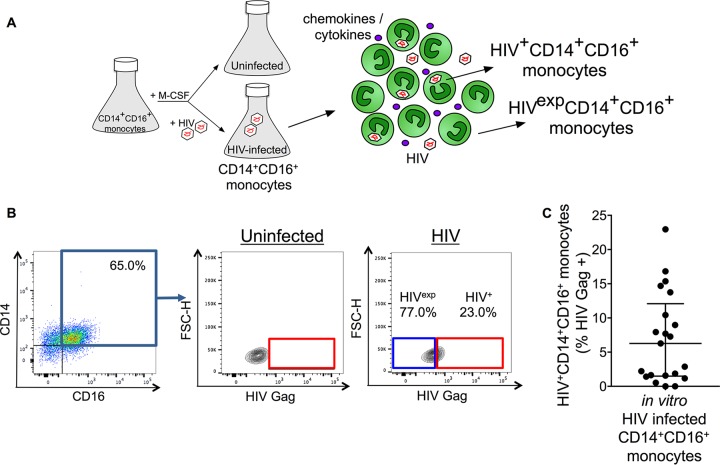
Detection of HIV^+^ and HIV^exp^ CD14^+^ CD16^+^ monocytes by flow cytometry. (A) Schematic of HIV^+^ and HIV^exp^ CD14^+^ CD16^+^ monocytes. Cultures enriched for mature CD14^+^ CD16^+^ monocytes are infected with HIV or left uninfected. The HIV-infected CD14^+^ CD16^+^ monocytes cultured nonadherently in Teflon-coated flasks are heterogeneous, as are cells in infected individuals, consisting of CD14^+^ CD16^+^ monocytes that are infected with HIV (HIV^+^ CD14^+^ CD16^+^ monocytes) and cells that are exposed to HIV, viral proteins, and inflammatory mediators but remain uninfected (HIV^exp^ CD14^+^ CD16^+^ monocytes). (B) Uninfected and HIV-infected mature CD14^+^ CD16^+^ monocytes are identified by CD14 and CD16 expression. After gating on the CD14^+^ CD16^+^ cell population, HIV Gag gating is determined based on negative signal of the uninfected cells. Gating is applied to HIV-infected cells. This determines the HIV^+^ and HIV^exp^ CD14^+^ CD16^+^ monocyte subsets. (C) Quantification of the percentage of HIV^+^ CD14^+^ CD16^+^ monocytes from 21 independent leukopak donors. Data are represented as mean ± standard error of the mean (SEM).

Myeloid cells and CD4^+^ T cells can be infected with HIV, which may lead to subsequent establishment of viral reservoirs in different tissue compartments, including the gut and CNS. Monocytes, cells of the myeloid lineage, have been shown to sustain replication-competent virus, even after suppressive ART (48). CD14^+^ CD16^+^ monocytes are the most susceptible monocytes to HIV infection, and a higher percentage of these cells with HIV DNA correlates with CNS disease in both human and macaque studies (42, 44, 45, 49). CD14^+^ CD16^+^ monocytes, like all other cells and tissues in infected individuals or cell cultures, are infected heterogeneously. A small percentage of the cells are infected with HIV, termed HIV^+^ CD14^+^ CD16^+^ monocytes, whereas the majority of cells from an infected individual or cell culture remain uninfected but are exposed to the virus, viral proteins, and inflammatory mediators that are present in the extracellular environment of those cells, termed HIV^exp^ CD14^+^ CD16^+^ monocytes (Fig. 1A). To determine the percentages of HIV^+^ and HIV^exp^ CD14^+^ CD16^+^ monocytes, we examined the intracellular expression of the HIV capsid protein Gag in cultured mature CD14^+^ CD16^+^ monocytes infected with the CCR5-tropic isolate HIV_ADA_.

To examine infection of cultured mature CD14^+^ CD16^+^ monocytes, we obtained high numbers of primary CD14^+^ CD16^+^ monocytes using a culture method we described previously (21, 49, 50). Briefly, CD14^+^ monocytes were obtained by CD14^+^ magnetic bead selection from peripheral blood mononuclear cells (PBMCs) of leukopaks from HIV-negative individuals. The CD14^+^ cells were cultured nonadherently in Teflon-coated flasks in the presence of macrophage colony-stimulating factor (M-CSF) for 2 to 3 days to obtain cultures enriched to on average 70 to 75% in mature CD14^+^ CD16^+^ monocytes (*n* > 300). The cultured mature CD14^+^ CD16^+^ monocytes were then left uninfected or were infected with HIV_ADA_ (1 μg/ml) for 8 h (at 10 × 10^6^ cells/ml). If infected, virus was spun off after 8 h, and viral replication enabled for an additional 64 h. If uninfected, cells were also spun after 8 h, and culture continued for another 64 h. Three days after infection, cells were examined for HIV Gag expression by flow cytometry. HIV Gag antibody specificity and the lower limit of detection of the antibody were determined first using uninfected (THP-1) and HIV-infected (strain 89.6) monocytic cells (THP89GFP). Uninfected THP-1 cells were confirmed as negative, and HIV Gag was detected accurately in infected THP89GFP cells at infection rates as low as 0.5% (see Fig. S1 in the supplemental material). Mature CD14^+^ CD16^+^ monocytes were stained for surface CD14 and CD16, fixed, permeabilized, and stained for HIV Gag (p24) (Fig. 1B). Cells were then analyzed by flow cytometry. We determined that 6.9% (range, 0.0 to 23.0%) of cells were HIV^+^ CD14^+^ CD16^+^ monocytes (Fig. 1C). The remaining 93.1% of the cells was considered HIV^exp^ CD14^+^ CD16^+^ monocytes. Uninfected CD14^+^ CD16^+^ monocytes obtained from the same leukopak donor, cultured in a separate flask, were used as a control for each experiment. Uninfected cells were HIV Gag negative (Fig. 1B). Isotype control antibody staining was negative as well. These data indicate that we specifically detected intracellular HIV Gag in the HIV^+^ CD14^+^ CD16^+^ monocytes, even at low infection levels, and that there was no false positivity detected in the HIV^exp^ CD14^+^ CD16^+^ monocytes.

10.1128/mBio.01280-17.1 FIG S1  Specific detection of HIV^+^ cells. (A to D) Uninfected THP-1 cells and HIV-infected THP89GFP cells were cultured in separate flasks and treated with tumor necrosis factor alpha (TNF-α [2 ng/ml]) for 72 h. Stimulation with TNF-α induces viral replication and concomitant GFP expression (FITC positivity) from the THP89GFP cells. The THP-1 and THP89GFP cells were mixed in different ratios just prior to staining for HIV Gag. (A) Schematic of THP-1 and THP89GFP cultures and of the preparation of different cell ratios for HIV Gag staining. (B) Flow cytometry plots of the FITC and HIV Gag expression of uninfected THP-1 cells after stimulation with TNF-α. (C) Flow cytometry plots of the FITC and HIV Gag expression of HIV-infected THP89GFFP cells after stimulation with TNF-α. (D) Table indicating the different ratios of cells and the percentage of cells that were FITC and HIV Gag positive in each fraction (representative example of *n =* 2). Download FIG S1, EPS file, 12.6 MB. Copyright © 2017 Veenstra et al. 2017 Veenstra et al. This content is distributed under the terms of the Creative Commons Attribution 4.0 International license.

### Junctional proteins are increased on HIV^+^ CD14^+^ CD16^+^ monocytes.

CD14^+^ CD16^+^ monocytes are key mediators of HIV neuropathogenesis (11, 12, 21, 37, 38, 46, 51–53). They transmigrate across the BBB in response to chemokines, including CCL2, which is one of the proposed mechanisms of viral entry into the CNS (21, 22, 54). The junctional proteins JAM-A and ALCAM are essential to CD14^+^ CD16^+^ monocyte transmigration across the BBB (21, 46), but it remains unclear whether surface JAM-A and ALCAM on these cells are changed due to direct infection or due to exposure and whether this mediates a difference in the BBB transmigration of the cells.

**FIG 2:**
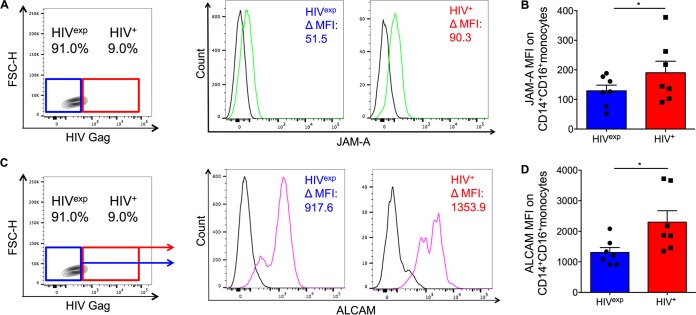
Junctional proteins JAM-A and ALCAM are increased on HIV^+^ CD14^+^ CD16^+^ monocytes. (A) Representative graph showing gating strategy on HIV^+^ and HIV^exp^ CD14^+^ CD16^+^ monocytes to examine JAM-A on both subsets. Representative histograms show JAM-A (green) and IgG1 isotype-matched control antibody (black) expression on HIV^+^ and HIV^exp^ CD14^+^ CD16^+^ monocytes. ΔMFI (change in mean fluorescence intensity) is calculated by subtracting JAM-A MFI from IgG1 isotype MFI. (B) Surface JAM-A on HIV^+^ and HIV^exp^ CD14^+^ CD16^+^ monocytes (*n =* 7). (C) Representative graph showing gating strategy on HIV^+^ and HIV^exp^ CD14^+^ CD16^+^ monocytes to examine ALCAM on both subsets. Representative histograms show ALCAM (pink) and IgG1 isotype-matched control antibody (black) expression on HIV^+^ and HIV^exp^ CD14^+^ CD16^+^ monocytes. ΔMFI is calculated by subtracting ALCAM MFI from IgG1 isotype MFI. (D) Surface ALCAM on HIV^+^ and HIV^exp^ CD14^+^ CD16^+^ monocytes (*n =* 7). Data are represented as mean ± SEM. Significance was determined by Wilcoxon’s signed-rank test. Significance is compared to baseline unless indicated otherwise. *, *P* < 0.05.

To assess whether infection changed the expression of junctional proteins essential to CD14^+^ CD16^+^ monocyte transmigration, we examined surface JAM-A and ALCAM on HIV^+^ and HIV^exp^ CD14^+^ CD16^+^ monocytes. Cultured HIV-infected mature CD14^+^ CD16^+^ monocytes were stained for CD14, CD16, JAM-A, or ALCAM, fixed, permeabilized, and stained for HIV Gag. HIV^+^ and HIV^exp^ CD14^+^ CD16^+^ monocytes were identified as described above (Fig. 1B). Surface JAM-A and ALCAM (change in mean fluorescent intensity [ΔMFI]) were determined by subtracting the IgG isotype-matched control MFI from the JAM-A or ALCAM MFI for both HIV^+^ and HIV^exp^ CD14^+^ CD16^+^ monocyte subsets (Fig. 2A and C). We found that HIV^+^ CD14^+^ CD16^+^ monocytes expressed significantly higher surface JAM-A (Fig. 2B [*P* < 0.05]) and ALCAM (Fig. 2D [*P* < 0.05]) than did HIV^exp^ CD14^+^ CD16^+^ monocytes.

### HIV^+^ CD14^+^ CD16^+^ monocytes preferentially transmigrate across the BBB.

To determine whether increased junctional proteins on HIV^+^ CD14^+^ CD16^+^ monocytes mediated a functional benefit to those cells, we assessed the BBB transmigration properties of HIV^+^ and HIV^exp^ CD14^+^ CD16^+^ monocytes. We used real-time PCR for HIV Gag to identify HIV^+^ CD14^+^ CD16^+^ monocytes as this method is highly sensitive and detects even those cells that are not undergoing active viral replication. The real-time PCR assay was established previously as a reliable method to detect HIV Gag in CD14^+^ CD16^+^ monocytes (55). We modified the assay by using HIV Gag primers to detect viral DNA and β-globin primers to detect genomic DNA and generated a standard curve with a plasmid for each quantitative PCR (qPCR) run as described in Materials and Methods. We were able to detect HIV Gag copies accurately with a limit of detection of 10 HIV DNA copies per 10^6^ cells.

**FIG 3:**
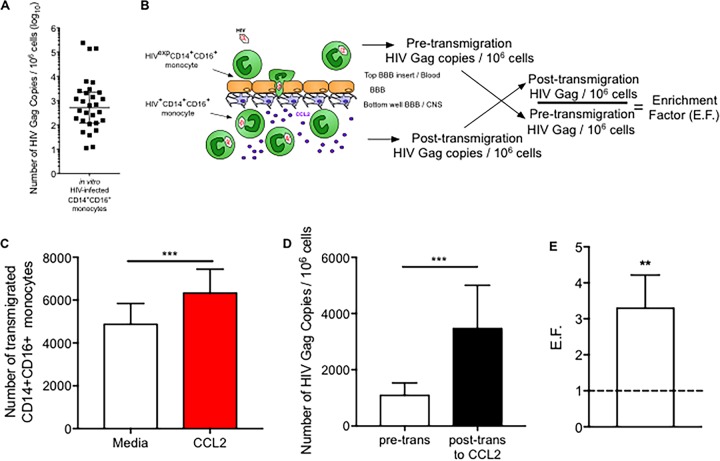
HIV^+^ CD14^+^ CD16^+^ monocytes preferentially transmigrate across the BBB. (A) Quantification of the number of HIV Gag copies per 10^6^ cells by qPCR. THP89GFP cells were used as a positive control, as these cells express one HIV Gag copy per cell. The number of HIV Gag copies from 10^6^ mature CD14^+^ CD16^+^ monocytes infected with HIV in culture was quantified (*n =* 30). (B) Schematic representation of transmigration across the human BBB tissue culture model. An EF was calculated by dividing the posttransmigration HIV Gag number per 10^6^ cells by the pretransmigration HIV Gag number. (C and D) HIV-infected mature monocytes, including both HIV^+^ and HIV^exp^ CD14^+^ CD16^+^ monocytes, were added to the top of the BBB and CCL2 to the bottom of the wells on the bottom side of the BBB. Cells were allowed to transmigrate, after which transmigrated cells from the bottom of the wells were collected and analyzed by flow cytometry and qPCR for HIV DNA. (C) HIV-infected mature monocytes from 13 independent leukopak cultures were allowed to transmigrate across the BBB for 16 h in response to medium (open bar) and CCL2 (red bar). The number of CD14^+^ CD16^+^ monocytes that transmigrated across the BBB was determined by flow cytometry. (D) The number of HIV Gag copies per 10^6^ cells was determined pretransmigration (pre-trans) for the 13 independent leukopak cultures that were also analyzed by flow cytometry, as well as for the cells that transmigrated across the BBB in response to CCL2 (posttransmigration [post-trans]). (E) The EF was calculated for the 13 independent leukopak cultures that were analyzed by the qPCR HIV DNA assay. Data are represented as mean ± SEM. Significance was determined by Wilcoxon’s signed-rank test. Significance is compared to baseline unless indicated otherwise. **, *P* < 0.01; ***, *P* < 0.001.

DNA was isolated from 10^6^ CD14^+^ CD16^+^ monocytes that were matured and HIV infected in culture as described in Materials and Methods. We determined that the median number of HIV Gag copies in this mixed population (HIV^+^ and HIV^exp^) was 510 per 10^6^ cells (range, 11 to 238,193 copies per 10^6^ cells, with 1 sample undetectable). THP89GFP cells with one HIV Gag copy per cell were used as a positive control (Fig. 3A). To determine whether HIV^+^ CD14^+^ CD16^+^ monocytes transmigrated preferentially, we added the mixed population of CD14^+^ CD16^+^ monocytes to the top of the BBB model and allowed the cells to transmigrate across the BBB to CCL2 (200 ng/ml) for 16 h. The number of HIV Gag DNA copies per 10^6^ cells was determined on a subset of the cells that were added to the BBB pretransmigration. After transmigration, cells were collected from the bottom of the wells, analyzed by flow cytometry to determine the number of cells that transmigrated, and analyzed by qPCR to determine the number of HIV Gag DNA copies per 10^6^ cells posttransmigration (Fig. 3B).

The heterogeneous population of HIV^+^ and HIV^exp^ CD14^+^ CD16^+^ monocytes transmigrated significantly more to CCL2 than to media (Fig. 3C [*P* < 0.0001]). The median number of HIV Gag copies per 10^6^ cells pretransmigration was 333 (interquartile range [IQR], 84 to 1,774). Posttransmigration the median number of HIV Gag copies per 10^6^ cells was significantly increased to 929 (IQR, 214 to 5,130) (Fig. 3D [*P* < 0.001]). Each experiment was performed with cells from a different leukopak donor, and outcomes varied widely as indicated by the IQR. Therefore, an enrichment factor (EF) for each experiment was calculated by dividing the number of HIV Gag copies per 10^6^ cells posttransmigration by the HIV Gag copy number per 10^6^ cells pretransmigration. We determined that the EF for cells that transmigrated across the BBB to CCL2 is 3.3. This is significantly higher than the pretransmigration HIV Gag copy number, set to an EF of 1 (Fig. 3E [*P* < 0.01]). This indicates that there is preferential transmigration of HIV^+^ CD14^+^ CD16^+^ monocytes across the BBB.

### Schematic of possible outcome for blocking experiments

**FIG 4:**
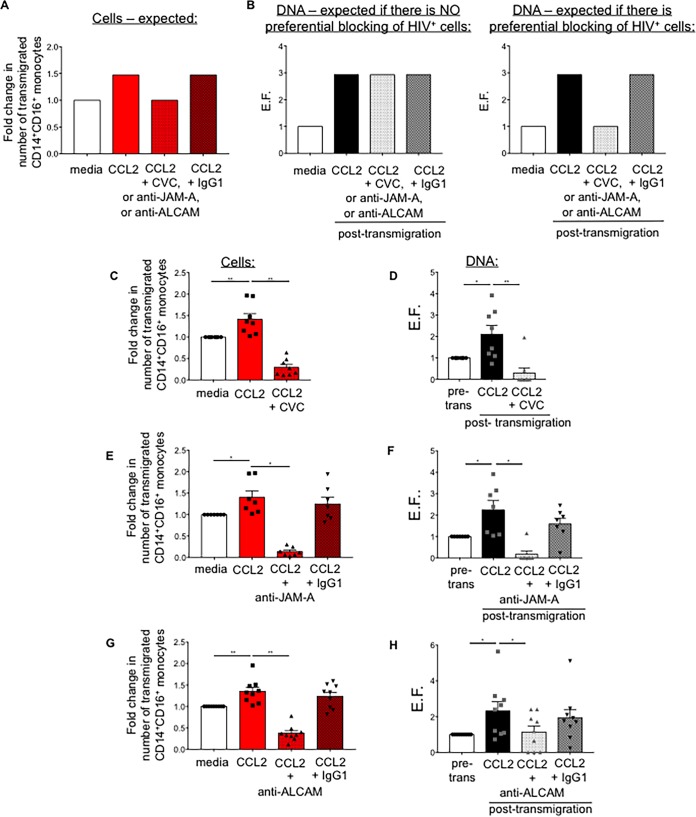
CCR2, JAM-A, and ALCAM are therapeutic targets to block and reduce preferential transmigration of HIV^+^ CD14^+^ CD16^+^ monocytes. (A to H) HIV-infected (both HIV^+^ and HIV^exp^) mature CD14^+^ CD16^+^ monocytes (1.5 × 10^5^) were added to the BBB model in the presence or absence of 100 nM CVC or 20 μg/ml anti-JAM-A or anti-ALCAM antibody or an IgG1 isotype control antibody. Cells were allowed to transmigrate in response to 200 ng/ml CCL2 for 16 h. A subset of CD14^+^ CD16^+^ monocytes was stained with CD14 and CD16 for analysis by flow cytometry pretransmigration, and DNA was collected from 10^6^ CD14^+^ CD16^+^ monocytes to quantify the number of HIV Gag copies pretransmigration. Transmigrated cells were collected from the bottom of 4 quadruplicate wells for flow cytometric analysis and transmigrated cells from 10 wells for qPCR HIV DNA analysis. Data are represented relative to baseline (media). (A) Schematic representation of expected transmigration results. The graph indicates the expectation of transmigration analyzed by flow cytometry, determining the number of HIV-infected (HIV^+^ and HIV^exp^) CD14^+^ CD16^+^ monocytes that transmigrated across the BBB. (B, left panel) Schematic representation of the expected results by qPCR HIV DNA assay if there is no preferential blocking of HIV^+^ CD14^+^ CD16^+^ monocytes by CVC or the anti-JAM-A or anti-ALCAM blocking antibodies. If there is no preferential blocking, the ratio of HIV^+^ and HIV^exp^ cells would be the same posttransmigration as pretransmigration, and all posttransmigration conditions would have the same EF. (B, right panel) Schematic representation of the expected results by qPCR HIV DNA assay if there is preferential blocking of HIV^+^ CD14^+^ CD16^+^ monocytes by CVC or the anti-JAM-A or anti-ALCAM blocking antibodies. Preferential blocking of HIV^+^ CD14^+^ CD16^+^ monocytes would mean there would be fewer or no HIV^+^ cells in the posttransmigration population of the blocking conditions, and the EF would be reduced. (C, E, and G) Quantification by flow cytometry of the number of CD14^+^ CD16^+^ monocytes that transmigrated across the BBB in the presence or absence of (C) CVC (*n =* 8), (E) anti-JAM-A or IgG1 isotype control antibody (*n =* 7), and (G) anti-ALCAM or IgG1 isotype control antibody (*n =* 9). (D, F, and H) qPCR HIV DNA analysis quantified the number of HIV Gag copies per 10^6^ CD14^+^ CD16^+^ monocytes pretransmigration (pre-trans) and posttransmigration. An EF was calculated for BBB transmigration assays with (D) CVC (*n =* 8), (F) anti-JAM-A antibody or IgG1 isotype control (*n =* 7), and (H) anti-ALCAM antibody or IgG1 isotype control (*n =* 9). Data are represented as mean ± SEM. Significance was determined by Wilcoxon’s signed-rank test. *, *P* < 0.05; **, *P* < 0.01; NS, not significant.

To examine whether CCR2, JAM-A, and ALCAM are potential targets to eliminate or reduce transmigration of HIV^+^ CD14^+^ CD16^+^ monocytes to CCL2, we added CVC (100 nM), a CCR2/CCR5 dual inhibitor (47), or either anti-JAM-A or anti-ALCAM blocking antibodies (both at 20 μg/ml) to cells transmigrating across the BBB. An IgG1 isotype-matched negative-control antibody was used as a control for the blocking antibodies. Cells were collected after transmigration and analyzed by flow cytometry for cell number and by qPCR for HIV DNA content. Examples of our expected results are indicated in Fig. 4A to C.

For cells transmigrating across the BBB, we expected that CVC would reduce transmigration of HIV-infected CD14^+^ CD16^+^ monocytes (comprised of both HIV^+^ and HIV^exp^ cells) to CCL2, compared with CCL2-mediated transmigration. We also expected that the anti-JAM-A and anti-ALCAM antibodies would significantly reduce transmigration of HIV-infected CD14^+^ CD16^+^ monocytes and that the IgG1 isotype control antibody would not differ significantly from CCL2-mediated transmigration (Fig. 4A), as described previously (21). If there is no preferential blocking of HIV^+^ and HIV^exp^ CD14^+^ CD16^+^ monocytes, the ratios of HIV^+^ and HIV^exp^ CD14^+^ CD16^+^ monocytes would be the same posttransmigration as pretransmigration, and the EFs would be equal for all conditions posttransmigration (Fig. 4B, left panel). This would also be the case when fewer cells transmigrated in the presence of CVC or anti-JAM-A or anti-ALCAM antibody, since the EF is calculated with the number of HIV Gag copies per 10^6^ cells and thus uses normalized values. However, if there is preferential blocking, there would be fewer or no HIV^+^ CD14^+^ CD16^+^ monocytes posttransmigration, and the EF would be reduced (Fig. 4B, right panel). We expected that the EF for cells treated with the isotype control antibody would be similar to that for non-antibody-treated cells that transmigrate to CCL2, as it should not affect their transmigration.

### CCR2, JAM-A, and ALCAM are therapeutic targets to block transmigration of HIV^+^ CD14^+^ CD16^+^ monocytes.

We determined that CCL2 significantly increased transmigration across the BBB of heterogeneously HIV-infected CD14^+^ CD16^+^ monocytes compared to media alone and that CVC, anti-JAM-A, and anti-ALCAM blocking antibodies significantly reduced CCL2-mediated transmigration of those cells. IgG1 isotype control antibody did not significantly reduce transmigration, indicating specific blocking (Fig. 4C, E, and G [*P* < 0.05]). The number of HIV Gag copies, as indicated by EF, was significantly increased posttransmigration to CCL2. CVC blocked transmigration of all HIV^+^ CD14^+^ CD16^+^ monocytes from 6 independent leukopak donors and reduced transmigration of those cells from 2 other leukopak donors. Overall CVC significantly reduced transmigration of HIV^+^ CD14^+^ CD16^+^ monocytes, from an EF of 2.1 to an EF of 0.3 (Fig. 4D [P < 0.01]). Antibodies against JAM-A blocked transmigration of all HIV^+^ CD14^+^ CD16^+^ monocytes from 6 independent leukopak donors and reduced transmigration of those cells from only 1 other leukopak donor. Overall anti-JAM-A significantly reduced transmigration of HIV^+^ CD14^+^ CD16^+^ monocytes from an EF of 2.2 to an EF of 0.2 (Fig. 4F [*P* < 0.05]). IgG1 isotype control antibody did not significantly reduce transmigration of HIV^+^ CD14^+^ CD16^+^ monocytes. Anti-ALCAM antibodies significantly reduced transmigration of HIV^+^ CD14^+^ CD16^+^ monocytes from an EF of 2.3 to an EF of 1.1 (Fig. 4H [*P* < 0.05]). It blocked transmigration of all HIV^+^ CD14^+^ CD16^+^ monocytes from 3 independent leukopak donors and reduced transmigration of those cells from 6 other leukopak donors. IgG1 isotype control antibody did not significantly reduce transmigration of HIV^+^ CD14^+^ CD16^+^ monocytes. These data indicate that anti-JAM-A antibody blocks completely transmigration of HIV^+^ CD14^+^ CD16^+^ monocytes in 86% of experiments, CVC in 75% of experiments, and ALCAM in 33% of experiments. Across experiments, transmigration of HIV^+^ CD14^+^ CD16^+^ monocytes was blocked completely in 7 out of 10 experiments. This suggests that CVC, as well as blocking antibodies to JAM-A, and potentially ALCAM, could significantly reduce viral entry into the CNS.

## DISCUSSION

The eradication of HIV in an effort to effect a cure is an extremely challenging task. The “shock and kill” approach is dependent upon the ability to induce reactivation of viral replication from viral reservoirs and to target the cells with actively replicating virus for elimination (8). HIV within the CNS is particularly difficult to target, since therapeutics may not adequately reach this tissue compartment, given its barriers and complex anatomy (56). Such strategies within the CNS could result in inflammation and further damage. Until a safe “shock and kill” strategy or vaccine is developed for an HIV cure, infection and seeding of tissue compartments such as the CNS need to be prevented and viral reseeding eliminated or reduced. In this study, we determined that JAM-A and ALCAM are essential to the preferential transmigration of HIV^+^ CD14^+^ CD16^+^ monocytes and that blocking antibodies against these junctional proteins may eliminate or significantly reduce the transmigration across the BBB specifically of these infected cells. In addition, we demonstrated that the CCR2/CCR5 dual inhibitor CVC preferentially blocks the transmigration of HIV^+^ CD14^+^ CD16^+^ monocytes. These data suggest that JAM-A, ALCAM, and CCR2 help mediate the preferential entry into the brain of HIV^+^ CD14^+^ CD16^+^ monocytes, leading to infection and viral seeding of the CNS and establishment and continued replenishment of viral reservoirs. The data also indicate that these junctional proteins and CCR2 are potential therapeutic targets and that addition to preexposure prophylaxis or current ART regimens of therapeutics that target those proteins may be essential in preventing viral entry and injury to the CNS.

The CNS is infected with HIV within 4 to 8 days after peripheral infection, and HIV can be detected within 1 to 2 weeks in infiltrating mononuclear cells in the perivascular and subpial spaces (13, 14, 39). Monocytes and macrophages in the CNS were shown to harbor HIV (9, 10). More specifically, the CD14^+^ CD16^+^ monocyte subset was shown to enter the CNS, and cells expressing CD14 and CD16 in HIV encephalitis brain tissue were found to contain HIV protein (11). Peripheral CD14^+^ CD16^+^ monocytes from people on ART preferentially harbor HIV compared to other monocyte subsets, and a higher number of CD14^+^ CD16^+^ monocytes carrying HIV DNA are present in the periphery of infected individuals who experience HIV-associated dementia (HAD) compared to those without HAD (42, 44, 45). However, transmigration into the CNS of infected HIV^+^ CD14^+^ CD16^+^ monocytes was still not well characterized, as it is very challenging to study this in humans or with primary human cells. Studies with a simian immunodeficiency virus (SIV) model showed that HIV^+^ cells from the periphery continue to enter the brain and that blocking leukocyte traffic with integrin antibodies decreases early infection of the CNS and neuronal injury later in HIV infection (12, 52). Future studies addressing this continued replenishment of HIV^+^ CD14^+^ CD16^+^ monocytes into the CNS of humans could include examining the cerebrospinal fluid (CSF) of infected subjects over time and analyzing the HIV envelope sequences for monocyte-specific cell surface markers. Such a study would complement our finding that primary human HIV^+^ CD14^+^ CD16^+^ monocytes preferentially transmigrate across the BBB. Our study suggests that early after peripheral infection, HIV^+^ CD14^+^ CD16^+^ monocytes preferentially enter the CNS and may release virus to infect the CNS, contributing to the establishment of viral reservoirs that mediate, in part, the neuronal damage and inflammation that lead to HIV neuropathogenesis.

We determined that HIV^+^ CD14^+^ CD16^+^ monocytes express increased JAM-A and ALCAM compared to the uninfected, but exposed HIV^exp^ CD14^+^ CD16^+^ monocyte subset. JAM-A and ALCAM are expressed by uninfected monocytes that are not exposed to virus, but to a similar or lesser extent than on HIV^exp^ CD14^+^ CD16^+^ monocytes (not shown). The junctional proteins JAM-A and ALCAM are expressed on many cells, including but not limited to most leukocytes, endothelial cells, and epithelial cells. Their expression is modulated by inflammatory factors that may lead to their redistribution to the cell surface of endothelial cells and potentially *de novo* synthesis or protein redistribution to the cell surface in monocytes (57, 58). Under other neuroinflammatory conditions, as shown with mouse models of cerebral ischemia and multiple sclerosis, the presence of these junctional proteins on BBB endothelial cells is reduced, which leads to loss of BBB integrity (59, 60). Continuous loss of BBB integrity has not been observed in HAND, but junctional proteins are essential to monocyte transmigration across the BBB because there is homotypic interaction with junctional proteins on the endothelial cells of the BBB (21, 58, 61). Unlike their significant role in monocyte transmigration, expression of JAM-A and ALCAM on neutrophils is relatively low, and on T cells they do not participate significantly in their transmigration (46). The data in our study suggest that the increased expression of these junctional proteins on HIV^+^ CD14^+^ CD16^+^ monocytes mediates their preferential transmigration. When we used antibodies against JAM-A and ALCAM, we reduced the number of CD14^+^ CD16^+^ monocytes that transmigrated across the BBB but also specifically targeted and reduced the transmigration of HIV^+^ CD14^+^ CD16^+^ monocytes. Anti-JAM-A antibodies were most effective, completely eliminating transmigration of HIV^+^ CD14^+^ CD16^+^ monocytes in 6 of 7 experiments.

CCR2 was previously shown by our laboratory to be increased on CD14^+^ CD16^+^ monocytes from HIV-infected individuals with HAND. In addition, we demonstrated that the CD14^+^ CD16^+^ monocytes from people with HAND transmigrate significantly more across the BBB to CCL2 than those from people without HAND (62). Moreover, CCR2 is increased on the mixed population of HIV-infected (HIV^+^ and HIV^exp^) CD14^+^ CD16^+^ monocytes compared to uninfected counterparts (21). This suggests that CCR2 plays a pivotal role in mediating HAND pathogenesis. CVC is a CCR2/CCR5 inhibitor recently tested in a clinical trial for HAND (NCT02128828) and if successful may be used in ART regimens. We therefore examined whether CVC was able to reduce or eliminate the preferential transmigration of HIV^+^ CD14^+^ CD16^+^ monocytes. We found that CVC significantly reduced transmigration of CD14^+^ CD16^+^ monocytes and prevented complete transmigration of HIV^+^ CD14^+^ CD16^+^ monocytes in 6 of 8 experiments. This suggests that CCR2 is an important therapeutic target to eliminate viral seeding of the CNS and reduce subsequent neuronal damage and neuroinflammation.

Infection of specific tissues and establishment and replenishment of viral reservoirs remain incompletely understood. In the ART era, and in suppressed individuals, it is even more difficult to determine the extent to which viral reservoirs mediate pathology and are able to contribute to new infections. However, recent studies have shown that viral reservoirs are not eliminated, even in individuals with complete suppression for many years and even while viral reservoirs decay over time (63–65). There may be several reasons why viral reservoirs remain, even though decay occurs. It could be that there is continuous replenishment of viral reservoirs (66). One mechanism by which this replenishment occurs could be by entry of infected cells, such as HIV^+^ CD14^+^ CD16^+^ monocytes, into tissues. This may be possible even in someone on suppressive ART, since HIV DNA remains present in peripheral blood cells of infected individuals on successful therapy (67, 68). Infection of progenitor cells or cells within the bone marrow may also contribute to reservoir replenishment (69–71). It may also be that viral reservoirs remain because there are areas of local active viral replication under suppressive ART, as shown in a study in lymphoid tissue (72). The fact that HIV compartmentalization occurs and that there is diversification of virus between the CNS and peripheral blood even in individuals on suppressive ART (73) may indicate that local active viral replication also occurs within the CNS. This could be due, in part, to ART not reaching parts of the brain parenchyma effectively—for example, due to a low CNS penetration effectiveness of certain ART regimens (74, 75). Our findings that HIV^+^ CD14^+^ CD16^+^ monocytes preferentially transmigrate across the BBB suggest that it is likely that this contributes to initial seeding and replenishment of reservoirs within the CNS. This is especially concerning since monocytes harbor infectious virus even after viral suppression by ART (48). Although we did not distinguish between cells with replication-competent or replication-defective virus in our flow cytometry and HIV DNA assays, even cells infected with replication-defective virus can drive pathogenesis. Release of replication-defective virus, for example, can mediate activation of surrounding cells, facilitating HIV infection of other (CNS) cells, while cells with replication-defective virus can also release early viral proteins that are neurotoxic, including HIV Tat (76, 77). Thus, the potential to prevent infectious replication-competent or replication-defective virus from entering the CNS through blocking the entry of HIV^+^ CD14^+^ CD16^+^ monocytes by targeting their JAM-A and CCR2, and potentially ALCAM, may be a new area to examine in aiming to eliminate viral seeding and replenishment, as well as to reduce the neuroinflammation and neuronal damage that contribute to cognitive deficits.

## MATERIALS AND METHODS

### Materials.

Cenicriviroc was received from Allergan (Parsippany-Troy-Hills, NJ). Anti-JAM-A (clone J10.4) was from Santa Cruz Biotechnology (Santa Cruz, CA) and anti-ALCAM (clone 81) from Antigenix America (Huntington Station, NY). Purified IgG1 isotype control antibody was a gift from Chemocentryx (Mountain View, CA). THP-1, a monocytic leukemia cell line, and THP-1 cells infected with HIV 89.6 that express enhanced green fluorescent protein (GFP) under the control of the HIV promoter (THP89GFP) such that fluorescence and viral replication are coupled, were a gift from Olaf Kutsch (University of Alabama, Birmingham, AL) and were described previously (62).

### Cell isolation, culture, and HIV infection.

Peripheral blood mononuclear cells (PBMCs) were isolated by Ficoll-Paque Plus (GE Healthcare, Uppsala, Sweden) density gradient centrifugation from anonymous leukopaks obtained from the New York Blood Center. Monocytes were isolated from PBMCs by magnetic bead-positive selection using the CD14 EasySep separation kit (Stem Cell Technologies, Vancouver, Canada). The freshly isolated CD14^+^ monocytes were then cultured nonadherently for 2 to 3 days in Teflon-coated flasks at 2 × 10^6^ cells/ml in supplemented RPMI in the presence of 10 ng/ml M-CSF (PeproTech, Rocky Hill, NJ) to facilitate monocyte maturation and yield “mature CD14^+^ CD16^+^ monocytes” as described previously (21, 49). These cells were then infected with HIV, and a subset from the same culture was left uninfected. For HIV infection, cells were resuspended to 10 × 10^6^ cells/ml in Teflon-coated flasks for nonadherent culture and inoculated with 1 μg/ml HIV_ADA_. After 8 h, virus was removed by centrifugation of the cells, and monocytes were resuspended to 2 × 10^6^ cells/ml in fresh medium with 10 ng/ml M-CSF and cultured nonadherently in Teflon-coated flasks for an additional 64 h to facilitate HIV replication and infection. For all HIV-infected monocyte cultures, supernatant levels of HIV p24 were measured using the high-sensitivity p24 AlphaLISA (PerkinElmer, Waltham, MA) to confirm infection. Supernatant HIV p24 levels ranged from 0.6 to 10.5 ng/ml.

### Flow cytometry.

Mature CD14^+^ CD16^+^ monocytes were stained using antibodies specific for human CD14 (BD; clone M5E2), and CD16 (BD; clone 3G8). Expression of the junctional proteins JAM-A and ALCAM was examined using JAM-A conjugated with fluorescein isothiocyanate (FITC) (BioLegend; clone OV-5B8; 0.5 μg), and biotinylated ALCAM (clone 105902, 0.25 μg [R&D]). Streptavidin-allophycocyanin (APC) (0.15 μg [EBioscience]) was used as a secondary reagent for biotinylated ALCAM; corresponding isotype-matched negative-control antibodies were also used. Titers of antibodies were obtained to determine optimal staining conditions. Cells (2 × 10^5^ in 100 μl) were stained in the dark on ice for 30 min, washed once, and fixed with 2% paraformaldehyde. To assess HIV infection and determine the percentages of HIV^+^ and HIV^exp^ CD14^+^ CD16^+^ monocytes, after staining for CD14, CD16, and JAM-A or ALCAM, cells were fixed with 2% paraformaldehyde for 20 min, permeabilized with 0.1% Triton X-100, in 1× phosphate-buffered saline (PBS) for 5 min, and stained in 100 μl 1% bovine serum albumin (BSA) (Thermo Fisher Scientific, Waltham, MA)–1× PBS with an antibody to HIV Gag (clone KC57; form, RD1 [Beckman-Coulter) at 1:40. At least 10,000 events were acquired with the BD FACSCantoII flow cytometer. Analysis was performed using FlowJo software (v.10.0.8 [Treestar, Ashland, OR]).

### qPCR HIV DNA assay.

Genomic DNA was isolated from mature CD14^+^ CD16^+^ monocytes using a QIAamp DNA blood minikit (Qiagen, MD) and stored at −20°C until use. HIV Gag content was analyzed using a previously described duplex real-time PCR method that demonstrated intraassay and interassay reproducibility (55). β-Globin primers and primers to HIV Gag (Integrated DNA Technologies, Inc., Coralville, IA), as well as probes for each (Thermo Fisher Scientific, Waltham, MA), were used to detect genomic DNA and proviral DNA, respectively (Table 1). A standard curve was generated for each independent assay with a plasmid containing β-globin and HIV Gag constructs. The standard curve was used to calculate relative HIV Gag DNA copy number per 10^6^ cells for all samples. THP89GFP cells, with a one copy of viral DNA per cell, were used as a positive control. Assays were run using the StepOne Plus thermocycler (Applied Biosystems, Foster City, CA).

**TABLE 1:** β-Globin and HIV Gag primers and probes used for the detection of genomic DNA and proviral DNA

Primer or probe^a^	Sequence (5′→3′)
β-Globin	
Forward primer	AGG GCC TCA CCA CCA ACT TC
Reverse primer	TCA CTA GCA ACC TCA AAC AGA CAC C
Probe (5′ VIC, 3′ TAMRA)	CTC CTG AGG AGA AGT CTG CCG TTA CTG CC
HIV Gag	
Forward primer	TCA GCC CAG AAG TAA TAC CCA TGT
Reverse primer	CAC TGT GTT TAG CAT GGT GTT T
Probe (5′ 6-FAM, 3′ TAMRA)	ATT ATC AGA AGG AGC CAC CCC ACA AGA

^a^ TAMRA, 6-carboxytetramethylrhodamine; 6-FAM, 6-carboxyfluorescein.

### Human BBB model.

The BBB model consists of human brain microvascular endothelial cells (BMVECs [Applied Cell Biology Research Institute, Kirkland, WA]) and human cortical astrocytes (obtained from the former Human Tissue Repository at Albert Einstein College of Medicine) cocultured on opposite sides of a gelatin-coated tissue culture insert with 3-μm pores (BD Falcon, Franklin Lakes, NJ), as described previously (20, 21, 78). The human cortical astrocytes used in the BBB model were isolated as part of an approved research protocol at the Albert Einstein College of Medicine. Astrocytes were seeded on the bottom (CNS side) of the insert and BMVECs added to the top (peripheral blood side). The cells were grown to confluence on the inserts for 3 days, at which time astrocytic foot processes penetrate the insert and contact the BMVEC layer to create a BBB impermeable to albumin.

### BBB transmigration assay.

Mature CD14^+^ CD16^+^ monocytes (1.5 × 10^5^) were added to the top of BBB coculture inserts (“peripheral” side) placed in wells of a 24-well plate. Medium or 200 ng/ml CCL2 (PeproTech) was added to the wells on the bottom side (“CNS” side) of the inserts. For BBB transmigration experiments that included CVC or the blocking antibodies, the mature CD14^+^ CD16^+^ monocytes and CVC (100 nM), anti-JAM-A, anti-ALCAM, or IgG1 antibodies (all at 20 μg/ml) were added concomitantly to the top of the BBB cocultures. Each transmigration condition was assayed with 4 replicate inserts for flow cytometry analysis and 10 replicate inserts for HIV DNA analysis. The transmigrated cells were collected from the bottom of the wells on the basolateral side of the inserts after 16 h of transmigration. For flow cytometry, CD14^+^ CD16^+^ monocytes were stained for human CD14 (clone M5E2 [BD]) and CD16 (clone 3G8 [BD]) and fixed with 2% paraformaldehyde, and the number of transmigrated cells was quantified by flow cytometry. For HIV DNA analysis, transmigrated cells were collected, spun, and stored at −80°C until DNA isolation and quantification.

To assess transmigration of HIV^+^ CD14^+^ CD16^+^ monocytes, we determined the number of HIV Gag copies per 10^6^ CD14^+^ CD16^+^ monocytes pretransmigration from a subset of the CD14^+^ CD16^+^ monocytes that were to be added to the BBB and determined the number of HIV Gag copies per 10^6^ CD14^+^ CD16^+^ monocytes posttransmigration. The number of HIV Gag copies for each was calculated as described above in the section “qPCR HIV DNA assay.” We used the acquired data to calculate an enrichment factor (EF) according to the formula EF = (posttransmigration HIV Gag/10^6^ cells)/(pretransmigration HIV Gag/10^6^ cells).

### Statistical analysis.

Statistical analyses were performed using Prism 6.0g (GraphPad Software, Inc., San Diego, CA). Mann-Whitney *U* tests were used for paired nonparametric measures and Wilcoxon’s test for nonpaired nonparametric measures to determine statistical significance (*P* < 0.05).

### Ethics statement.

Leukopaks were obtained from the New York Blood Center, New York, NY. All leukopaks received were anonymized. Institutional Review Board (IRB) approval for these studies was obtained from the Einstein Human Research Protection Program (HRPP) at the Albert Einstein College of Medicine (IRB no. 1994-003).
